# A kinetic analysis of four high velocity, horizontally focused step‐up variations for acceleration training

**DOI:** 10.1002/ejsc.12150

**Published:** 2024-06-17

**Authors:** Daniel J. Glassbrook, Chris A. Dorman, Tim L.A. Doyle, Jarrod A. Wade, Joel T. Fuller

**Affiliations:** ^1^ Faculty of Medicine, Health and Human Sciences Department of Health Sciences Macquarie University Sydney New South Wales Australia; ^2^ South Sydney Rabbitohs Rugby League Club Sydney New South Wales Australia

**Keywords:** force, impulse, rate of force development, strength training, velocity

## Abstract

Step‐up variations are frequently used in sports performance to develop coordinated and powerful movements that transfer to running. This study aimed to quantify the kinetic characteristics of the first foot contact of four different step‐up variations. Ten professional rugby league players participated in this study and performed the Barbell One Box Step‐Up with Catch (BB1), Barbell Two Box Step‐Up (BB2), Vest Two Box Run (VEST) and Step‐Up Jump (JUMP) as part of routine in‐season strength training sessions during one season. Peak force, total impulse and maximal rate of force development (RFD) were measured from first foot contact on the step‐up box. Significantly greater peak force and RFD were observed in JUMP than any other variation (standardized mean difference; SMD: 3.9–5.5; *p* < 0.001). Total impulse was equal between JUMP and BB1, and significantly greater in JUMP than BB2 and VEST (SMD: 1.3–2.3; *p* < 0.001), and in BB1 than BB2 and VEST (SMD: 1.8–2.8; *p* < 0.001). Significantly larger peak force and RFD were observed in BB2 and VEST than BB1 (SMD: 0.6–0.7) and in total impulse in BB2 than VEST (SMD: 1.6) (*p* < 0.05). The results of this study highlight that step‐up exercise variations maximize different kinetic characteristics, which may transfer differently to athlete running performance.

## Highlights

- The Step‐Up Jump variation was shown to produce the largest relative peak force, largest rate of force development (RFD) and equal largest total impulse and therefore may have the greatest transferability to maximal speed running through the maximization of the stance phase.
- The Barbell Two Box Step‐Up (BB2) and Vest Two Box Run (VEST), produced significantly greater peak relative force and RFD than the Barbell One Box Step‐Up with Catch (BB1) and therefore may have better transferability to acceleration ability.
- Coaches can use the findings of this study to assist their selection of step‐up variations that are best suited to develop running ability and should consider the testing results and individual requirements of each athlete.

## 1 INTRODUCTION

Running is a key component of many team sports and enhancing an athlete's ability to run at high velocities is a key role of a strength and conditioning coach. The step‐up is a unilateral resistance exercise that may satisfy the principles of specificity (Lambert et al., 2008, p. 8) and overload (Lambert et al., 2008, pp. 6–7) for strength and conditioning coaches who need to prepare athletes for various forms of running performance (Bosch et al., 2005, 2015). Indeed, step‐up variations are frequently used in sports performance (Appleby, Cormack, & Newton, 2019; Bosch et al., 2005, 2015), clinical (Bailey et al., 2015; Ebert et al., 2017; Ross, 1997) and fitness industry (Fujarczuk et al., 2006; Rutkowska‐Kucharska et al., 2017) settings to develop coordinated and powerful movements that transfer to running (Brearley et al., 2019). In sport context, running ability can be further broken down into acceleration ability and maximal running speed, and players are often required to utilise these capacities to make it to a position nearby or sprint over a distance (Duthie et al., 2003). Acceleration is the ability to generate speed from a stationary or moving start, and acceleration performance is strongly dependent on the first steps (Frost et al., 2011), where the ability to develop high force and time taken to do so is paramount for speed generation (Lockie et al., 2011; Weyand et al., 2000). Producing large amounts of force during short ground‐contact durations is also a hallmark of fast maximal running speed ability (Weyand et al., 2000). This can be investigated through ground reaction forces (GRF) (Nagahara et al., 2018), which are broken down into anterior‐posterior (GRF_A‐P_), medio‐lateral (GRF_M‐L_) and vertical (GRF_Vert_) force directions; combining these three axes into a vector quantity is referred to as a resultant (GRF_Resultant_). GRF_A‐P_ is particularly relevant for acceleration and GRF_Vert_ is particularly relevant for maximal running speed (Nagahara et al., 2018; von Lieres Und Wilkau et al., 2020). Some studies have shown no significant correlation between acceleration performance and GRF_Vert_ (Colyer et al., 2018; Rabita et al., 2015) and other studies have shown that smaller GRF_Vert_ are beneficial to initial acceleration performance (Nagahara et al., 2018, 2021). During the initial acceleration phase, GRF_Vert_ needs to be large enough to lift the bodies centre of mass (CoM) and create an appropriate flight time to prepare for the next foot strike phase, although if GRF_Vert_ is too large during this phase it may negatively affect acceleration performance (Weyand et al., 2000). Instead, peak GRF_Vert_ appears to be important in the later stages of acceleration, where large peak GRF_Vert_ likely indicates better performance in this stage (Nagahara et al., 2021). Additionally, the overall GRF_Resultant_ vector magnitude is increased by a large GRF_Vert_ which is likely to benefit acceleration performance (Morin et al., 2011). Horizontal step‐up exercise variations, such as those used in the present study may be used to develop both GRF_Vert_ and GRF_A‐P_ and therefore develop running mechanics, as both vertical and anterior displacement is required to move from the floor to atop a box and the horizontal element is emphasized through these techniques. Hip strength in both the anterior and posterior chain are crucial to fast running ability (Rodriguez et al., 2020; Schache et al., 2014). Traditional step‐up's are performed by the participant starting with a square stance, and then stepping up to a box and ending with both feet atop the same box; they are a knee dominant exercise focussing on vertical expression of force. Further emphasis on the horizontal expression of force and the use and development of hip musculature can be added by starting with a hip hinge (trunk flexion) and a split stance, or through step‐up variations that incorporate a second box and additional anterior displacement.

Only one study to date has quantified the kinetics of a step‐up variation and reported concentric force and impulse for 70%–90% one repetition maximum (1RM) in well trained rugby players (Appleby, Newton, & Cormack, 2019). The results of this study determined that when compared to back‐squats, the loaded step‐up exercise produced larger peak and average GRF_Resultant_ per leg than the back‐squat (ES: 1.45–2.70). These results suggest that the step‐up is applicable to lower limb maximal strength development. These results are also supported by Appleby, Cormack, & Newton, (2019) who showed similar benefits of both exercises, but did not measure kinetic variables such as GRF. Notably, the step‐up variations used in these studies are largely vertical in nature and did not include a high velocity, or horizontal expression of force. Therefore, it can be argued that these variations don't emphasise anterior‐posterior, or horizontal displacement sufficiently to impact linear acceleration. Instead, to impact linear acceleration, step‐up variations should include a greater horizontal expression of GRF. There are several variations of step‐up exercises but a lack of research into what variation is optimal for different and specific situations. Load may be applied to the step‐up for example via barbell, dumbbell, or weighted vest, or the complexity of the exercise increased by the addition of a second box and a greater horizontal propulsion requirement (Bosch et al., 2015). The loading strategy applied to the step‐up exercise will not only affect the amount of load that may be applied (e.g., a larger load is typically possible via barbell, than by dumbbell or weighted vest), but will also influence the athletes stability as their CoM is raised or lowered depending on the loading strategy applied, that is, greater instability may be created by a barbell than a weighted vest or dumbbells due a raised CoM and extension of mass away from the body. An increased load requires increased force produced to create motion, however, an increase in the instability will impede force production. The majority of current step‐up research has utilized a simple forward step‐up technique and in athletic populations load applied via barbell (Appleby, Cormack, & Newton, 2019; Appleby, Newton, & Cormack, 2019), with some research in non‐athletic populations applying load via weighted vest (Salem et al., 2004; Shaw et al., 1998). Some research has also sought to quantify variables such as electromyographic activity of specific muscles and GRF, of the simple step‐up (step aerobics), without load and in non‐athletic populations (Ayotte et al., 2007; Chinkulprasert et al., 2011; Fujarczuk et al., 2006; Rutkowska‐Kucharska et al., 2017). Despite this relatively broad research of step‐ups, there is little known about the kinetic differences in step‐up variations in athletic populations and therefore the present study will address a sizeable gap in the literature.

The step‐up exercise and each of its variations can be incorporated across a range of cycles within a periodized strength training plan. Plyometric boxes made out of metal or wood are prevalent in high performance gyms and can range in height from 10 cm (4”) to 76.2 cm (30”). The step‐up exercise is performed to a plyometric box at a height that facilitates a 90° knee flexion angle at foot contact (typically, 30–42 cm high) to replicate the motion of running and knee drive (Appleby, Cormack, & Newton, 2019; Appleby, Newton, & Cormack, 2019; Appleby et al., 2020). The step‐up may be of particular relevance to running based team sports, where an increase in lower body strength using the step‐up has been identified. Lower body strength is important for running, as strength is the ability to create force. Appleby, Cormack, & Newton, (2019) trained the step‐up and back‐squat independently in developmental rugby players for eight weeks using loads of up to 88% of 1RM and demonstrated similar strength benefits for each exercise (step‐up, ES: 0.79 ± 0.40; back‐squat, ES: 0.63 ± 0.17). In a subsequent study (Appleby et al., 2020), the same authors and with the same step‐up and back‐squat training protocol showed small and variable improvements in 5 m (ES: −0.37 ± 0.41) and 20 m (ES: −0.31 ± 0.31) sprint time and 50° change of direction (COD, ES: −0.50 ± 0.54) test performance in developmental rugby players with the step‐up exercise. In another study, Worrell et al. (1993) demonstrated in healthy university physical therapy students that a four‐week training program of the lateral step‐up, using an additional 25% of bodyweight as external load, increased performance in a single leg hop test for distance and a single leg hop test for time (*p* ≤ 0.05). These tests reflect lower body power and function, but the authors did not directly measure and report force, velocity, or power data. Considering the potential sport‐specific benefits of the step‐up exercise for running based sports, the force and power data of step‐up variations may be of particular interest to strength and conditioning coaches and provide a means to create overload of a specific movement pattern within a periodized strength program.

Loaded step‐up variations appear to provide a unique stimulus for strength and conditioning coaches to use in athletic preparation, due to their potential to satisfy principles of specificity (Lambert et al., 2008, p. 8), coordination (19, p. 5) and transfer training to running. However, there is a paucity of current scientific quantification of the kinetics of the step‐up exercise and a kinetic profile of high velocity, horizontally orientated step‐up variations has not yet been done. This information will be important to help substantiate the widespread use of step‐up exercises in running based sport. Therefore, the purpose of this paper is to quantify the kinetic characteristics of the first foot contact of four different high velocity, horizontally focused step‐up variations.

## 2 MATERIALS AND METHODS

### 2.1 Experimental approach to the problem

In order to quantify the kinetic characteristics of the first foot contact during different step‐up variations, four step‐up variations were investigated as part of routine in‐season strength training sessions during one National Rugby League (NRL) season. Testing of each step‐up variation was performed on different days. Four step‐up exercise variations were implemented to generate different forces, movement patterns and movement velocities, including variations to test the ability to produce large GRF_Vert_ and direct it anteriorly (horizontally; propulsive). The four step up variations used in this study were, the Barbell One Box Step‐Up with Catch (BB1), Barbell Two Box Step‐Up (BB2), Vest Two Box Run (VEST) and Step‐Up Jump (JUMP). This study was conducted in accordance with the Declaration of Helsinki and was approved by the Macquarie University Human Research Ethics Committee (protocol number: 5201955829441). Written informed consent was obtained from all participants.

### 2.2 Participants

Ten professional rugby league players from one NRL club participated in this study (Age: 24.9 ± 2.7 years; Mass: 108.2 ± 8.8 kg; Height: 1.90 ± 0.06 m). Each participant played in the forwards position group and were free of any injuries that may impede step‐up ability. As this study was conducted through the course of the home and away fixtures, game to game turnaround affected the frequency of strength training sessions. Nine of the 10 participants completed at least one testing session for all four step‐up variations. One participant only completed testing for the BB1, BB2 and VEST variations, due to an injury sustained as part of their regular training and could not complete the JUMP on the intended testing day.

### 2.3 Procedures

Testing was undertaken during the in‐season period as part of each player's prescribed training program. The step‐up was included in the lower‐body strength session of the training week (only one variation) and the place of this session in the week varied in line with the weekly turn around dictated by the match schedule (matches are played Thursday to Sunday weekly). Participants completed at least one testing session for each step‐up variation and in the case that they completed more than one testing session for the variation, the result of the testing session with highest force value was used for analysis. The order of variant testing was (1) VEST, (2) BB2, (3) BB1 and (4) JUMP. The step‐up exercise was performed as the first lower body exercise in the prescribed strength program on the given testing day to ensure athletes were not fatigued and testing took place under the supervision of qualified strength and conditioning coaches. Participants were familiarized with all four variations of the step‐up (for illustrations, see Glassbrook et al., 2023) through regular inclusion of each variation, with varying loads, as part of their usual strength training sessions during the 16‐week pre‐season. The loads used in this study were determined by the head strength and conditioning coach, with respect to the individual requirements (i.e., task constraints) and techniques of each step‐up variation and to maximise the intended training stimulus of each exercise.

BB1 was performed with a loaded Olympic barbell held on the participants' shoulders. Three barbell loads were used (determined by head strength and conditioning coach), warm up: 60 kg, 80 kg and test set: 90 kg. From a slightly hip hinged (trunk flexion) static split stance position, the participant propels out of this position and uses one leg to drive into the wooden plyometric box (41.5 cm total height; 15 cm force plate plus 30 cm box) and stand up as quickly as possible. This box height was selected to replicate knee joint running mechanics (Bosch et al., 2005, 2015) and is consistent with box heights used in previous step‐up studies (Appleby, Cormack, & Newton, 2019; Appleby, Newton, & Cormack, 2019; Appleby et al., 2020). Once the leading leg has completed the step‐up to the first box the non‐leading leg is brought up to catch the body's momentum by stepping onto a second wooden plyometric box (61.0 cm). A 61.0 cm (24”) plyometric box is common place in high performance gyms. The movement finishes with the leading leg on top of the initial box and the non‐leading leg on top of the second box (Glassbrook et al., 2023). The participant then reverses the movement to un‐mount the two boxes and performs a second repetition, before changing the leading leg. The overall objective of the BB1 is to focus on and facilitate one explosive drive at first foot contact into the box, making the movement relatively specific to the first step of accelerating during sprinting. The BB1 was considered a high load and high force variant.

BB2 was performed with a loaded Olympic barbell held on the participants' shoulders. Four barbell loads were used (determined by head strength and conditioning coach), warm up: 40 kg, 50 kg, 60 kg and test set: 70 kg. The start of the BB2 is the same as the BB1, but then involves the non‐leading leg cycling through to step up and be driven down on the same wooden plyometric box (41.5 cm total height) as the BB1, then the non‐leading leg cycles through to step up and is and driven into a second wooden box (61.0 cm). Once the non‐leading leg has completed the step up, the leading leg is brought up so the participant completes the movement standing atop the second box (Glassbrook et al., 2023). The participant then reverses the movement to un‐mount the two boxes and performs a second repetition, before changing the leading leg. Because of the nature of this variant, it was considered a high load, speed strength variant. Due to the more dynamic and complex nature of the BB2 compared to the BB1 and the less stable finish position in the BB2, lower loads than with the BB1 were perceived to be more manageable while coordinating the movement.

VEST was performed with the participants wearing a weighted vest. Three vests of varying loads were used, warm up: 10 kg, 20 kg and test set: 30 kg. From a slightly hip hinged (trunk flexion) static split stance position the participant runs up the two wooden plyometric boxes, 41.5 cm (total height) and 61.0 cm. At the apex of the movement, the athlete uses their hands to stop their momentum by making contact with and gripping onto handles within the lifting rack to halt the participants' momentum. The participant finishes in an acceleration type posture, rear leg extended, toe off on the second box and front knee up (Glassbrook et al., 2023). This exercise was considered a low load, speed variant.

The JUMP was performed without any external load, that is, body‐mass load only. The JUMP is performed by the participant standing behind the wooden plyometric box (41.5 total height) in the same split stance hinged starting position as the other variations used in this study. The participant then lifts the lead leg and drives down forcefully into the box and propels themselves off the box into the air (Glassbrook et al., 2023). The participant lands back in the starting position alongside the box. Two repetitions are completed in this manner, before the leading leg is swapped and another two repetitions completed. This exercise was considered a body‐mass power variant.

A calibrated uniaxial 400‐series portable force plate (Fitness Technology, Adelaide, Australia) sampling at 600 Hz was placed under the step‐up box when testing each of the four variations of the exercise, therefore the height remained constant. The height of the force plate combined with the height of a 30 cm wooden step‐up box totaled 41.5 cm. The force plate was used to measure the peak vertical force (N), total impulse (N⋅s) and maximal RFD (N⋅s^−1^) from first foot contact on the box. Each of the three variables are able to be reliably measured by force plates (peak vertical force: ICC = 0.99; total impulse: ICC = 0.79; RFD: ICC = 0.94) (Rago et al., 2018; Souza et al., 2020). Data were collected in Ballistic Measurement System (Innervations, West Perth, Australia) and each repetition was trimmed to include only the initial first foot contact to toe off for each repetition. Raw data were then exported to Microsoft Excel, where peak values were extracted for each repetition. Data from the set with the highest barbell or vest load was used for analysis, where external load was added to the participant. Data were normalised to system mass (relative) by dividing the kinetic value by the system mass (sum of participant mass and external load) (Kipp et al., 2012; Loturco et al., 2021). All participants were experienced with the force plate through routine use.

### 2.4 Statistical analyses

All statistical analysis was performed in SPSS (v25, IBM, NY, USA) with statistical significance set at alpha equals 0.05. The dependent variables were peak force, maximum RFD and total impulse from the heaviest repetition performed by each athlete. Mixed models were used to compare the dependent variables between the four step‐up variants. Fixed effects were step‐up variant (4 levels), left versus right leg (2 levels) and session number (2 levels). Participant ID was considered a random effect with a variance components covariance structure. Residual plots were used to assess normality, homoscedasticity and independence of residuals. Pairwise comparisons for significant main effects were made based on estimated marginal means using least significant difference post hoc tests. Standardized mean differences (SMD) with 95% confidence intervals were calculated to determine the magnitude and consistency of pairwise differences. SMD magnitude was considered trivial (≤0.20), small (>0.2), moderate (>0.6), large (>1.2) and very large (>2.0) (Hopkins et al., 2009). G*Power (v. 3.1.9.7) analysis determined that the sample size of 10 participants could detect an effect size of *f* ≥ 0.4 across the four step‐up conditions with 80% power and an alpha of 0.05.

## 3 RESULTS

There was no significant effect of leg on any of the variables and thus results are presented as an average response across legs for each of the four step‐up variations and as mean ± standard deviation. Raw data are presented in Table 1.

**TABLE 1:** Raw data.

Variable	BB1	BB2	VEST	JUMP
Normalised/Relative				
Peak force (N/kg)	23.3 ± 7.3	27.8 ± 7.3	28.7 ± 7.3	63.5 ± 7.2
Total impulse (N⋅s/kg)	31.7 ± 3.7	25.0 ± 3.7	21.1 ± 3.7	29.6 ± 3.7
Maximal RFD (N⋅s^−1^/kg)	754.5 ± 271.8	927.8 ± 271.8	950.6 ± 271.8	2006.5 ± 265.8
Absolute				
Peak force (N)	4619.1 ± 1056.4	4967.1 ± 1056.4	3972.3 ± 1056.4	6780.6 ± 1027.41
Total impulse (N⋅s)	6288.2 ± 639.2	4437.6 ± 639.2	2929.6 ± 639.2	3178.0 ± 628.7
Maximal RFD (N⋅s^−1^)	149,723.1 ± 38,884.9	165,564.7 ± 38,884.9	131,627.9 ± 38,884.9	214,561.5 ± 37,812.3

*Note*: Data are presented as mean ± standard deviation.

Abbreviations: BB1, barbell one box step‐up with catch; BB2, barbell two box Step‐Up; JUMP, Step‐Up Jump; kg, kilograms.; N, newtons; N⋅s, newtons per second; N⋅s‐1, newtons per second squared; RFD, rate of force development; VEST, vest two box run.

**FIGURE 1:**
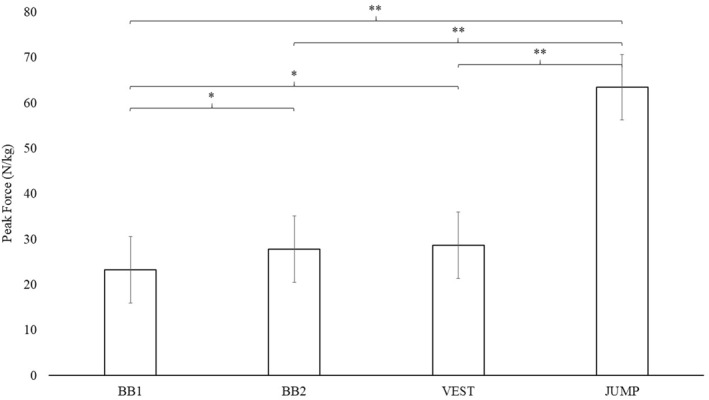
Normalised peak force results for all four step‐up variations. Data are presented as mean ± standard deviation. BB1, Barbell One Box Step‐Up with Catch; BB2, Barbell Two Box Step‐Up; VEST, Vest Two Box Run; JUMP, Step‐Up Jump; N, Newtons; kg, Kilogrammes; *, *p* < 0.05; **, *p* < 0.001. Note: for exact *p* values, refer to the results section.

Significantly larger relative peak force (N/kg) was observed in JUMP than BB1 (*p* < 0.001; SMD: 5.5; 95%CI: 4.9,6.2), BB2 (*p* < 0.001; SMD: 4.9; 95%CI: 4.3,5.5) and VEST (*p* < 0.001; SMD: 4.8; 95%CI: 4.2,5.4), BB2 than BB1 (*p* = 0.042; SMD: 0.6; 95%CI: 0.02,1.2) and VEST than BB1 (*p* = 0.016; SMD: 0.7; 95%CI: 0.1,1.3) (Figure 1).

**FIGURE 2:**
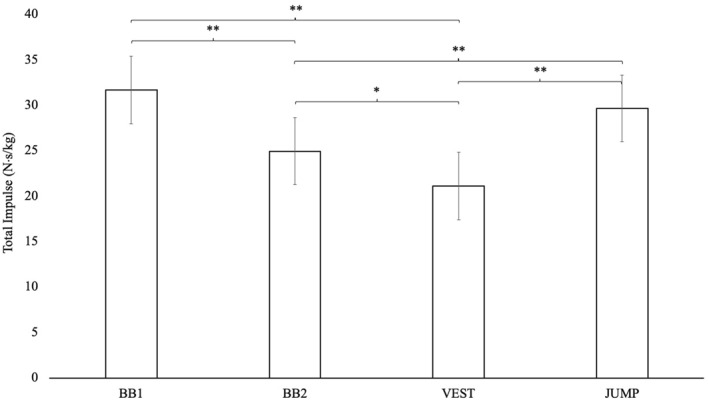
Normalised total impulse results for all four step‐up variations. Data are presented as mean ± standard deviation. BB1, Barbell One Box Step‐Up with Catch; BB2, Barbell Two Box Step‐Up; VEST, Vest Two Box Run; JUMP, Step‐Up Jump; N⋅s, Newtons per second; kg, Kilograms; *, *p* < 0.05; **, *p* < 0.001. Note: for exact *p* values, refer to the results section.

Significantly larger relative total impulse (N⋅s/kg) was observed in JUMP than BB2 (*p* < 0.001; SMD: 1.3; 95%CI: 0.6,2.0) and VEST (*p* < 0.001; SMD: 2.3; 95%CI: 1.6,3.0), in BB1 than BB2 (*p* < 0.001; SMD: 1.8; 95%CI: 1.1,2.5) and VEST (*p* < 0.001; SMD: 2.8; 95%CI: 2.2,3.2) and BB2 than VEST (*p* = 0.003; SMD: 1.6; 95%CI: 0.5,2.6) (Figure 2).

**FIGURE 3:**
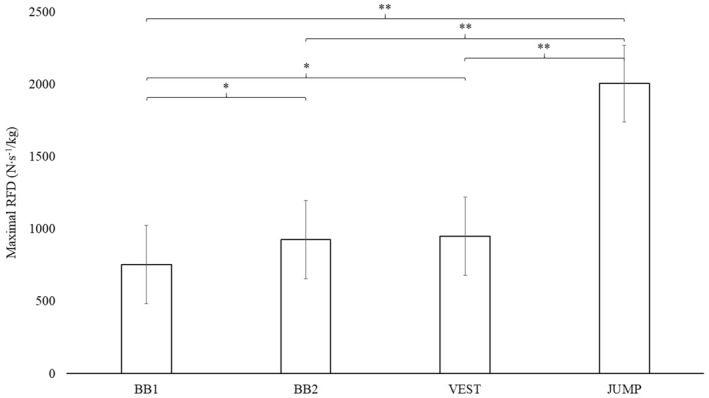
Normalised maximal rate of force development results for all four step‐up variations. Data are presented as mean ± standard deviation. BB1, Barbell One Box Step‐Up with Catch; BB2, Barbell Two Box Step‐Up; VEST, Vest Two Box Run; JUMP, Step‐Up Jump; RFD, Rate of Force Development; N⋅s^−1^, Newtons per second squared; kg, Kilograms; *, *p* < 0.05; **, *p* < 0.001. Note: for exact *p* values, refer to the results section.

Significantly larger relative maximal RFD (N⋅s^−1^/kg) was observed in JUMP than BB1 (*p* < 0.001; SMD: 4.7; 95%CI: 4.0,5.3), BB2 (*p* < 0.001; SMD: 4.0; 95%CI: 3.4,4.6) and VEST (*p* < 0.001; SMD: 3.9; 95%CI: 3.3,4.6), BB2 than BB1 (*p* = 0.037; SMD: 0.6; 95%CI: 0.04,1.2) and VEST than BB1 (*p* = 0.019; SMD: 0.7; 95%CI: 0.1,1.3) (Figure 3).

## 4 DISCUSSION

The ability of athletes to accelerate quickly and achieve high running speed is strongly linked to force production during their first steps. The purpose of this paper was to quantify the kinetic characteristics of the first foot contact of four different step‐up variations. The results of this study highlight a significant effect of step‐up variation on each measured kinetic variable. The main findings of the study were (1) performing a step‐up with a jump produced significantly greater peak relative force and RFD than any other step‐up variation and equal greatest total relative impulse, (2) peak relative force and RFD were significantly greater when stepping up to a second box and performing a step‐up wearing a weighted vest than the step‐up to a single box with a catch, (3) stepping up to a second box produces significantly larger total relative impulse than a step‐up wearing a weighted vest and (4) a step‐up to a single box with a catch produces significantly larger total relative impulse than stepping up to a second box and a step‐up wearing a loaded vest.

When training athletes who participate in running based sports, the results of this study suggest that the JUMP step‐up variation has the greatest ability to maximize the relative force at the foot‐ground interface, during the stance phase of the movement and potentially transfer to maximal running speed (where high vertical forces are beneficial). Interestingly, even though no external load was added to participants in this variation, the largest relative forces, equal largest relative total impulses and largest relative RFDs were observed. This can be explained by the powerful and ballistic nature of the movement and the force required to propel the body upwards into the air. Transfer of strength and skill is dependent on matching of the mechanics of each movement, such as contraction type, contraction velocity and joint angle (Wilson et al., 1996). Although vertical and not horizontal in nature (except for the initial movement from a split stance), the JUMP may still transfer to running performance due to similarities in contraction type, contraction velocity and joint angles and the unilateral nature of the movement. A hallmark of fast running performance is placing high force into the ground rapidly, at the stance phase (Weyand et al., 2000). The results of this study show the JUMP to be the best at maximizing a key kinetic attribute in force during the stance phase of maximal speed running.

The second key finding of this study showed a significantly greater peak relative force and RFD in the BB2 and VEST than the BB1. The VEST is designed as a low load, speed variant and the BB2 is considered to be a high load, speed strength variant. In contrast, the BB1 was considered a high load and force variant. Contrary to the intended high force nature of the BB1, it produced the lowest relative force of any step‐up variation and the third lowest absolute force behind the JUMP and BB2. This may be due to the slower nature of the movement, caused by performing the movement with a greater external load than in the VEST and BB2 variations. The increased mass limits the ability of athletes to accelerate quickly during the movement which impacts peak force production. This is exemplified by the concurrently larger RFD in the VEST and BB2 than BB1 step‐up variation. Additionally, the greater external load used for the BB1 compared to VEST and BB2 may have created instability in the movement by raising the CoM of the system, which in turn will affect force production potential. It may be the case that the participants were overloaded in this study when performing the BB1. If high relative force is the desired outcome for step‐up exercise prescription and variation selection, then practitioners should avoid slow speed, high load variations such as the BB1. Instead, the BB2 or VEST appear to be step‐up variations with better transfer to acceleration and maximal running speed performance.

The third and fourth key findings of this paper are regarding total relative impulse differences across step‐up variations. In contrast to findings for peak relative force and RFD, the BB1 produced significantly larger total relative impulse than the BB2 and VEST and similar values to the JUMP. As impulse is force multiplied by time, a large impulse can be explained by these variables. The BB1 is a slower movement compared to the VEST and BB2 on account of the large external load, and therefore there is a longer contact time on the force plate during the movement which equates to a larger total impulse; although contact time was not measured in this study. Significantly larger peak relative force was observed in BB2 and VEST than the BB1 and therefore a larger total impulse in BB1 than the BB2 and VEST can only be due to an increase in time. The larger contact time in the BB1 than BB2 and VEST could be due to the technique differences across the variations. The BB2 and VEST variations both conclude atop a second box, without any contact with the first box and associated force plate. The BB1 on the other hand concludes with the leading leg in contact with the first box and the non‐leading leg in contact with the second box, before reversing the movement. Although each repetition was trimmed to isolate from the initial first foot contact to toe off for each repetition, the technique of the BB1 likely lends itself to longer contact time during the step, than the BB2 and VEST.

The results of this study provide a kinetic profile and an indication of the kinetic differences at first foot contact for four different step‐up variations, which is highly applicable to general running focused conditioning. Coaches can use these findings to select step‐up variations that are best suited to develop running ability. When doing so, coaches should consider the testing results and individual requirements of each athlete. The JUMP step‐up variation was shown to produce the largest relative peak force and RFD and therefore may have the greatest transferability to maximal running speed ability through the maximization of the stance phase of running and production of high vertical forces quickly. Athletes seeking to improve their acceleration ability may benefit from the inclusion of step‐up variations that are horizontal in nature and create high vertical forces that are directed anteriorly (by moving forward in the exercise), such as the BB2 or VEST. Regardless, each step‐up variation should transfer to general running improvements via sufficient overload and specificity to running motion. A potential limitation of this study was that the data relates to only 10 athletes from only one Australian NRL club and therefore may not be representative of all rugby league athletes, such as those competing in other professional competitions (e.g., English Super League) or at an amateur or semi‐professional level, or other team sports where acceleration performance is important (e.g., soccer, rugby union, hockey). Sprint speed, or acceleration performance was not measured in this study, so the direct relationship between the present results and sprinting ability is speculative. Only vertical force was measured and no shear forces due to equipment availability; considering that all step‐up variations have a forward progression element, shear forces may provide additional insight into each step‐up variation. Different absolute loads were used across step‐up variations and these different loads may have contributed to the observed variance in kinetic outcomes, alongside the exercise modes. However, these differences were based on prescriptions by a highly trained strength and conditioning coach to maximize the intended training stimulus of each exercise. This is highly reflective of applied settings where jump‐ and speed‐based exercise variations will typically have less external weight than slower, non‐jump variants. Although data in this study were collected from a relatively small sample size as is necessary with elite sport and with limitations on variables and equipment availability, these limitations may be counteracted by the value of collecting data in a high‐performance population such as that used in this study, in comparison to other commonly used populations (e.g., sport science students) (Hecksteden et al., 2022; Skorski et al., 2021). Ground contact time was also not analysed in this study, future research may seek to analyse this variable which may provide further insight into the transference of step‐up variations to sprint stride kinetics. Finally, the results of this study are based on biomechanical responses to a single session rather than a training intervention that leads to the greatest running performance long‐term. Future research is warranted to investigate this topic.

In conclusion, there are several different variations of the step‐up exercise which can be used in strength and conditioning practice. The results of this study address a sizeable gap in the literature and highlight that step‐up exercise variations maximize different kinetic characteristics in comparison to each other; which may transfer differently to athlete performance. Of the four step‐up variations tested in this study, the results suggest that the JUMP may be the overall best variation for transfer to maximal speed running and the BB2 or VEST for acceleration. However, coaches should always consider the testing results and individual requirements of each athlete before selecting specific exercises.

## CONFLICT OF INTEREST STATEMENT

The authors report there are no competing interests to declare.

## References

[ejsc12150-bib-0001] Appleby, B. B. , S. J. Cormack , and R. U. Newton . 2019. “Specificity and Transfer of Lower‐Body Strength: Influence of Bilateral or Unilateral Lower‐Body Resistance Training.” The Journal of Strength & Conditioning Research 33(2): 318–326. 10.1519/jsc.0000000000002923. 30688873

[ejsc12150-bib-0002] Appleby, B. B. , S. J. Cormack , and R. U. Newton . 2020. “Unilateral and Bilateral Lower‐Body Resistance Training Does Not Transfer Equally to Sprint and Change of Direction Performance.” The Journal of Strength & Conditioning Research 34(1): 54–64. 10.1519/jsc.0000000000003035. 30844983

[ejsc12150-bib-0003] Appleby, B. B. , R. U. Newton , and S. J. Cormack . 2019. “Kinetics and Kinematics of the Squat and Step‐Up in Well‐Trained Rugby Players.” The Journal of Strength & Conditioning Research 33(1): S36–S44. 10.1519/jsc.0000000000003055. 30707142

[ejsc12150-bib-0004] Ayotte, N. W. , D. M. Stetts , G. Keenan , and E. H. Greenway . 2007. “Electromyographical Analysis of Selected Lower Extremity Muscles during 5 Unilateral Weight‐Bearing Exercises.” Journal of Orthopaedic & Sports Physical Therapy 37(2): 48–55. 10.2519/jospt.2007.2354. 17366959

[ejsc12150-bib-0005] Bailey, C. A. , and P. A. Costigan . 2015. “An Accelerometer as an Alternative to a Force Plate for the Step‐Up‐And‐Over Test.” Journal of Applied Biomechanics 31(6): 504–506. 10.1123/jab.2014-0214. 26157105

[ejsc12150-bib-0006] Bosch, F. , and K. Cook . 2015. Strength Training and Coordination: An Integrative Approach. 2010 Publishers Rotterdam.

[ejsc12150-bib-0007] Bosch, F. , and K. Klomp . 2005. Biomechanics and Exercise Physiology Applied in Practice. Elsevier Churchill Livingstone.

[ejsc12150-bib-0008] Brearley, S. , and C. Bishop . 2019. “Transfer of Training: How Specific Should We Be?” Strength and Conditioning Journal 41(3): 97–109. 10.1519/ssc.0000000000000450.

[ejsc12150-bib-0009] Chinkulprasert, C. , R. Vachalathiti , and C. M. Powers . 2011. “Patellofemoral Joint Forces and Stress during Forward Step‐Up, Lateral Step‐Up, and Forward Step‐Down Exercises.” Journal of Orthopaedic & Sports Physical Therapy 41(4): 241–248. 10.2519/jospt.2011.3408. 21289449

[ejsc12150-bib-0010] Colyer, S. L. , R. Nagahara , and A. I. T. Salo . 2018. “Kinetic Demands of Sprinting Shift across the Acceleration Phase: Novel Analysis of Entire Force Waveforms.” Scandinavian Journal of Medicine & Science in Sports 28(7): 1784–1792. 10.1111/sms.13093. 29630747

[ejsc12150-bib-0011] Duthie, G. , D. Pyne , and S. Hooper . 2003. “Applied Physiology and Game Analysis of Rugby Union.” Sports Medicine 33(13): 973–991. 10.2165/00007256-200333130-00003. 14606925

[ejsc12150-bib-0012] Ebert, J. R. , P. K. Edwards , D. P. Fick , and G. C. Janes . 2017. “A Systematic Review of Rehabilitation Exercises to Progressively Load the Gluteus Medius.” Journal of Sport Rehabilitation 26(5): 418–436. 10.1123/jsr.2016-0088. 27632888

[ejsc12150-bib-0013] Frost, D. M. , and J. B. Cronin . 2011. “Stepping Back to Improve Sprint Performance: a Kinetic Analysis of the First Step Forwards.” The Journal of Strength & Conditioning Research 25(10): 2721–2728. 10.1519/jsc.0b013e31820d9ff6. 21912339

[ejsc12150-bib-0014] Fujarczuk, K. , S. Winiarski , and A. Rutkowska‐Kucharska . 2006. “Ground Reaction Forces in Step Aerobics.” Acta of Bioengineering and Biomechanics 8. 19739592

[ejsc12150-bib-0015] Glassbrook, D. J. , C. A. Dorman , T. L. A. Doyle , J. A. Wade , and J. T. Fuller . 2023. Step‐Up Variations. figshare. 10.1002/ejsc.12150 PMC11295083 38886980

[ejsc12150-bib-0016] Hecksteden, A. , S. Forster , F. Egger , F. Buder , R. Kellner , and T. Meyer . 2022. “Dwarfs on the Shoulders of Giants: Bayesian Analysis with Informative Priors in Elite Sports Research and Decision Making.” Front Sports Act Living 4: 793603. 10.3389/fspor.2022.793603. 35368412 PMC8970347

[ejsc12150-bib-0017] Hopkins, William G. , Stephen W. Marshall , Alan M. Batterham , and Juri Hanin . 2009. “Progressive Statistics for Studies in Sports Medicine and Exercise Science.” Medicine & Science in Sports & Exercise 41(1): 3–12. 10.1249/mss.0b013e31818cb278. 19092709

[ejsc12150-bib-0018] Kipp, K. , J. Redden , M. B. Sabick , and C. Harris . 2012. “Weightlifting Performance Is Related to Kinematic and Kinetic Patterns of the Hip and Knee Joints.” The Journal of Strength & Conditioning Research 26(7): 1838–1844. 10.1519/jsc.0b013e318239c1d2. 21986692

[ejsc12150-bib-0019] Lambert, M. I. , W. Viljoen , A. Bosch , A. J. Pearce , and M. Sayers . 2008. “General Principles of Training.” In Olympic Textbook of Medicine in Sport, edited by M. P. Schwellnus , 1–48.

[ejsc12150-bib-0020] Lockie, R. G. , A. J. Murphy , T. J. Knight , and X. A. K. Janse de Jonge . 2011. “Factors that Differentiate Acceleration Ability in Field Sport Athletes.” The Journal of Strength & Conditioning Research 25(10): 2704–2714. 10.1519/jsc.0b013e31820d9f17. 21878822

[ejsc12150-bib-0021] Loturco, I. , L. A. Pereira , T. T. Freitas , C. Bishop , F. Pareja‐Blanco , and M. R. McGuigan . 2021. “Maximum Strength, Relative Strength, and Strength Deficit: Relationships with Performance and Differences between Elite Sprinters and Professional Rugby Union Players.” International Journal of Sports Physiology and Performance 16(8): 1148–1153. 10.1123/ijspp.2020-0342. 33588376

[ejsc12150-bib-0022] Morin, J.‐B. , P. Edouard , and P. Samozino . 2011. “Technical Ability of Force Application as a Determinant Factor of Sprint Performance.” Medicine & Science in Sports & Exercise 43(9): 1680–1688. 10.1249/mss.0b013e318216ea37. 21364480

[ejsc12150-bib-0023] Nagahara, R. , H. Kanehisa , A. Matsuo , and T. Fukunaga . 2021. “Are Peak Ground Reaction Forces Related to Better Sprint Acceleration Performance?” Sports Biomechanics 20(3): 360–369. 10.1080/14763141.2018.1560494. 30676878

[ejsc12150-bib-0024] Nagahara, R. , M. Mizutani , A. Matsuo , H. Kanehisa , and T. Fukunaga . 2018. “Association of Sprint Performance with Ground Reaction Forces during Acceleration and Maximal Speed Phases in a Single Sprint.” Journal of Applied Biomechanics 34(2): 104–110. 10.1123/jab.2016-0356. 28952906

[ejsc12150-bib-0025] Rabita, G. , S. Dorel , J. Slawinski , E. Sàez‐de‐Villarreal , A. Couturier , P. Samozino , and J.‐b. Morin . 2015. “Sprint Mechanics in World‐Class Athletes: a New Insight into the Limits of Human Locomotion.” Scandinavian Journal of Medicine & Science in Sports 25(5): 583–594. 10.1111/sms.12389. 25640466

[ejsc12150-bib-0026] Rago, V. , J. Brito , P. Figueiredo , T. Carvalho , T. Fernandes , P. Fonseca , and A. Rebelo . 2018. “Countermovement Jump Analysis Using Different Portable Devices: Implications for Field Testing.” Sports 6(3): 91. 10.3390/sports6030091. 30200384 PMC6162675

[ejsc12150-bib-0027] Rodriguez, M. W. , S. A. Menhennett , C. N. Vannatta , and T. W. Kernozek . 2020. “Relationship Among Maximum Hip Isometric Strength, Hip Kinematics, and Peak Gluteal Muscle Force during Running.” Physical Therapy in Sport 45: 188–196. 10.1016/j.ptsp.2020.06.009. 32827794

[ejsc12150-bib-0028] Ross, C. M. 1997. “Test‐retest Reliability of the Lateral Step‐Up Test in Young Adult Healthy Subjects.” Journal of Orthopaedic & Sports Physical Therapy 25(2): 128–132. 10.2519/jospt.1997.25.2.128. 9007771

[ejsc12150-bib-0029] Rutkowska‐Kucharska, A. , K. Wysocka , S. Winiarski , A. Szpala , and M. Sobera . 2017. “An Investigation into the Relation between the Technique of Movement and Overload in Step Aerobics.” Applied Bionics and Biomechanics 2017: 1–7. 10.1155/2017/3954907. PMC5350317 28348501

[ejsc12150-bib-0030] Salem, G. J. , S. P. Flanagan , M.‐Y. Wang , J.‐E. Song , S. P. Azen , and G. A. Greendale . 2004. “Lower‐Extremity Kinetic Response to Weighted‐Vest Resistance during Stepping Exercise in Older Adults.” Journal of Applied Biomechanics 20(3): 260–274. 10.1123/jab.20.3.260.

[ejsc12150-bib-0031] Schache, A. G. , T. W. Dorn , G. P. Williams , N. A. T. Brown , and M. G. Pandy . 2014. “Lower‐limb Muscular Strategies for Increasing Running Speed.” Journal of Orthopaedic & Sports Physical Therapy 44(10): 813–824. 10.2519/jospt.2014.5433. 25103134

[ejsc12150-bib-0032] Shaw, J. M. , and C. M. Snow . 1998. “Weighted Vest Exercise Improves Indices of Fall Risk in Older Women.” The Journals of Gerontology 53A: M53‐M58 53A(1): M53–M58. 10.1093/gerona/53a.1.m53. 9467434

[ejsc12150-bib-0033] Skorski, S. , and A. Hecksteden . 2021. “Coping with the “Small Sample–Small Relevant Effects” Dilemma in Elite Sport Research.” International Journal of Sports Physiology and Performance 16(11): 1559–1560. 10.1123/ijspp.2021-0467. 34653960

[ejsc12150-bib-0034] Souza, A. A. , M. Bottaro , V. A. Rocha , et al. 2020. “Reliability and Test‐Retest Agreement of Mechanical Variables Obtained during Countermovement Jump.” International Journal of Exercise Science 13: 6–17. 32148630 10.70252/XQXF8049 PMC7039490

[ejsc12150-bib-0035] von Lieres Und Wilkau, H. C. , N. E. Bezodis , J.‐B. Morin , G. Irwin , S. Simpson , and I. N. Bezodis . 2020. “The Importance of Duration and Magnitude of Force Application to Sprint Performance during the Initial Acceleration, Transition and Maximal Velocity Phases.” Journal of Sports Sciences 38(20): 2359–2366. 10.1080/02640414.2020.1785193. 32627681

[ejsc12150-bib-0036] Weyand, P. G. , D. B. Sternlight , M. J. Bellizzi , and S. Wright . 2000. “Faster Top Running Speeds Are Achieved with Greater Ground Forces Not More Rapid Leg Movements.” Journal of Applied Physiology 89(5): 1991–1999. 10.1152/jappl.2000.89.5.1991. 11053354

[ejsc12150-bib-0037] Wilson, G. J. , A. J. Murphy , and A. Walshe . 1996. “The Specificity of Strength Training: the Effect of Posture.” European Journal of Applied Physiology and Occupational Physiology 73(3–4): 346–352. 10.1007/bf02425497. 8781867

[ejsc12150-bib-0038] Worrell, T. W. , B. Borchert , K. Erner , J. Fritz , and P. Leerar . 1993. “Effect of a Lateral Step‐Up Exercise Protocol on Quadriceps and Lower Extremity Performance.” Journal of Orthopaedic & Sports Physical Therapy 18(6): 646–653. 10.2519/jospt.1993.18.6.646. 8281177

